# Diptera and *Drosophila* Karyotype Databases: A Useful Dataset to Guide Evolutionary and Genomic Studies

**DOI:** 10.3389/fevo.2022.832378

**Published:** 2022-03-17

**Authors:** Magnolia W. Morelli, Heath Blackmon, Carl E. Hjelmen

**Affiliations:** 1 Department of Biology, Utah Valley University, Orem, UT, United States,; 2 Department of Biology, Texas A&M University, College Station, TX, United States

**Keywords:** Diptera, *Drosophila*, karyotype, sex chromosome, chromosome number

## Abstract

Karyotypes and chromosome data have been widely used in many subfields of biology over the last century. Unfortunately, this data is largely scattered among hundreds of articles, books, and theses, many of which are only available behind paywalls. This creates a barrier to new researchers wishing to use this data, especially those from smaller institutions or in countries lacking institutional access to much of the scientific literature. We solved this problem by building two datasets for true flies (Order: Diptera and one specific to *Drosophila*), These datasets are available via a public interactive database that allows users to explore, visualize and download all data. The Diptera karyotype databases currently contain a total of 3,474 karyotype records from 538 publications. Synthesizing this data, we show several groups are of particular interest for future investigations by whole genome sequencing.

## INTRODUCTION

For more than a century, karyotypes have been used to determine chromosome number within species (Metz, 1914), in addition to identifying sex chromosome systems, aneuploidy events, and structural mutations of species or populations (Powell, 1997; The Tree of Sex Consortium, 2014; Blackmon et al., 2017). Chromosome number is a simple and fundamental piece of genomic information collected for a species. Understanding the mode and tempo of chromosome number evolution across the tree of life is an active area of research (Blackmon et al., 2019; Ruckman et al., 2020; Sylvester et al., 2020). Additionally, a major application of chromosome number is used to measure the quality and “completeness” of genome assemblies; better assemblies should have contig/scaffold numbers close to the haploid number of chromosomes for the species. In the case of sex chromosome systems, karyotyping can reveal the presence of heteromorphic sex chromosome systems (XX/XY, ZZ/ZW, X0, Z0, and multiple/complex), and be used to identify recent chromosomal changes, such as the formation of a neo-sex chromosome system through the fusion of an autosome to the ancestral sex chromosomes. Despite the importance of karyotype data for basic and applied research in the life sciences, this data remains largely scattered among hundreds of articles, books, and theses, many of which are only available behind paywalls.

Karyotype data in the insect order Diptera has been abundantly produced over the last century, due to the presence of easily visualized and differentiated polytene chromosomes (Metz, 1914, 1916; Painter, 1933; Painter, 1934; Koltzoff, 1934; Ward, 1949). In fact, polytene chromosomes were key to *Drosophila melanogaster* becoming one of the most widely used and important model organisms in genetics and evolutionary biology (Powell, 1997; Zhimulev et al., 2004). The banding patterns of these chromosomes allowed early researchers to make genetic maps, identify mutations within species, and investigate how populations diverge (Dobzhansky, 1946; Powell et al., 1972; Anderson et al., 1975). Polytene chromosomes are not only confined to *Drosophila*, but are documented throughout the entire order of Diptera, leading to many studies of polytene chromosomes in Culicidae (mosquitos), Chironomidae (midges), and Simuliidae (black flies) (Kitzmiller, 1963; Grossbach, 1977; Rothfels, 1979; Rao and Rai, 1987; Michailova et al., 2001; Adler et al., 2004; Petrova and Zhirov, 2014). Extensive karyotype work, has shown that Diptera have conserved (syntenic) regions, often referred to as Muller elements A–F, which suggest conservation of gene groupings and linkage (White, 1949; The Tree of Sex Consortium, 2014; Vicoso and Bachtrog, 2015). As these six elements are shown to be highly conserved in many species, it has been hypothesized that most species would not have haploid chromosome numbers which exceed six (Sved et al., 2016). This karyotype data has been reported in books, peer-reviewed journals, and proceedings. Unfortunately, a substantial number of these are not available online, and some are not in print today.

In this study, we describe a new database containing karyotype information for the insect order Diptera, in addition to a separate database for the *Drosophila* genus. A broad understanding of karyotype evolution in Diptera may reveal key species to answer fundamental questions about genome biology, and result in species to be prioritized for sequencing. A freely accessible database for this order, similar to others compiled in Coleoptera, Polyneoptera, and Amphibians (Blackmon and Demuth, 2015a; Perkins et al., 2019; Sylvester et al., 2020) would accelerate future research in chromosome evolution and provide more equitable access to data.

## MATERIALS AND METHODS

### Collection of Karyotypes

We compiled karyotype information for Diptera using both online and library sources. Initially, we compiled karyotype information from previous compilations of *Drosophila* data (Ward, 1949; Patterson and Stone, 1952; Clayton and Wasserman, 1957; King, 1975; Clayton, 1986). As these sources were not readily available online, we utilized the Texas A&M University Library system,^1^ University of Texas Library system,^2^ interlibrary loans, and the personal library of J. Spencer Johnston (Professor of Entomology, Texas A&M University). These records were supplemented with records collected by the Tree of Sex Consortium (^3^The Tree of Sex Consortium, 2014). Subsequently, we compiled records using Google Scholar searches combining Order (Diptera) or family names (for a total of 51 identified families) with “chromosome,” “chromosome number,” “karyotype,” “sex chromosome,” “Muller elements,” and “polytene chromosomes.” Additionally, we collected records by forward and backward citation tracking of previous compilations (Patterson and Stone, 1952; King, 1975).

References for records were compiled and condensed into one record for cases in which multiple reports are referring to the same data collection event. From each of the records obtained, we collected taxonomic information, chromosome number, chromosome morphology, sex system, and references (Table 1). Because *Drosophila* species have disproportionately more records than other families/genera and a different chromosome shape naming system, we created a separate database solely for this genus. While *Drosophila* species have their own database, their chromosome number data is also located in the more inclusive “Diptera Karyotype Database.”

### Development of Online Database

We used the R package Shiny version 1.7.1 to create a dynamic and interactive database (Chang et al., 2021). The framework of this database is based on a data repository (all of the collected karyotype and taxonomic information) and three user modules. The first user module allows a researcher to narrow down the data of interest based on taxonomic rank (Family and Genus). The second module allows users to visualize their data; in this module users can choose from the variables of haploid number, sex chromosome system, family and genus. The website will build plots of the chosen variables using a framework built on ggplot2 version 3.3.5 functions (Wickham et al., 2016). After visualizing the data users can select the third module which will display a table of all selected data. This module also allows the user to download a CSV file for offline use in their own research. Both the Diptera Karyotype Database and *Drosophila* Karyotype Database are hosted at www.karyotype.org with yearly updates, pending new records.

## RESULTS AND DISCUSSION

The current database contains 3,474 dipteran karyotypes which we compiled from 538 publications. While we did a thorough search of the literature, we have certainly not located all available data. Some papers are not easily located through internet searches or libraries, and/or may be in languages the authors were not comfortable translating. Users producing new records or who are aware of records missing from the database are encouraged to contact the authors to allow continued growth of this community resource.

**FIGURE 1 |:**
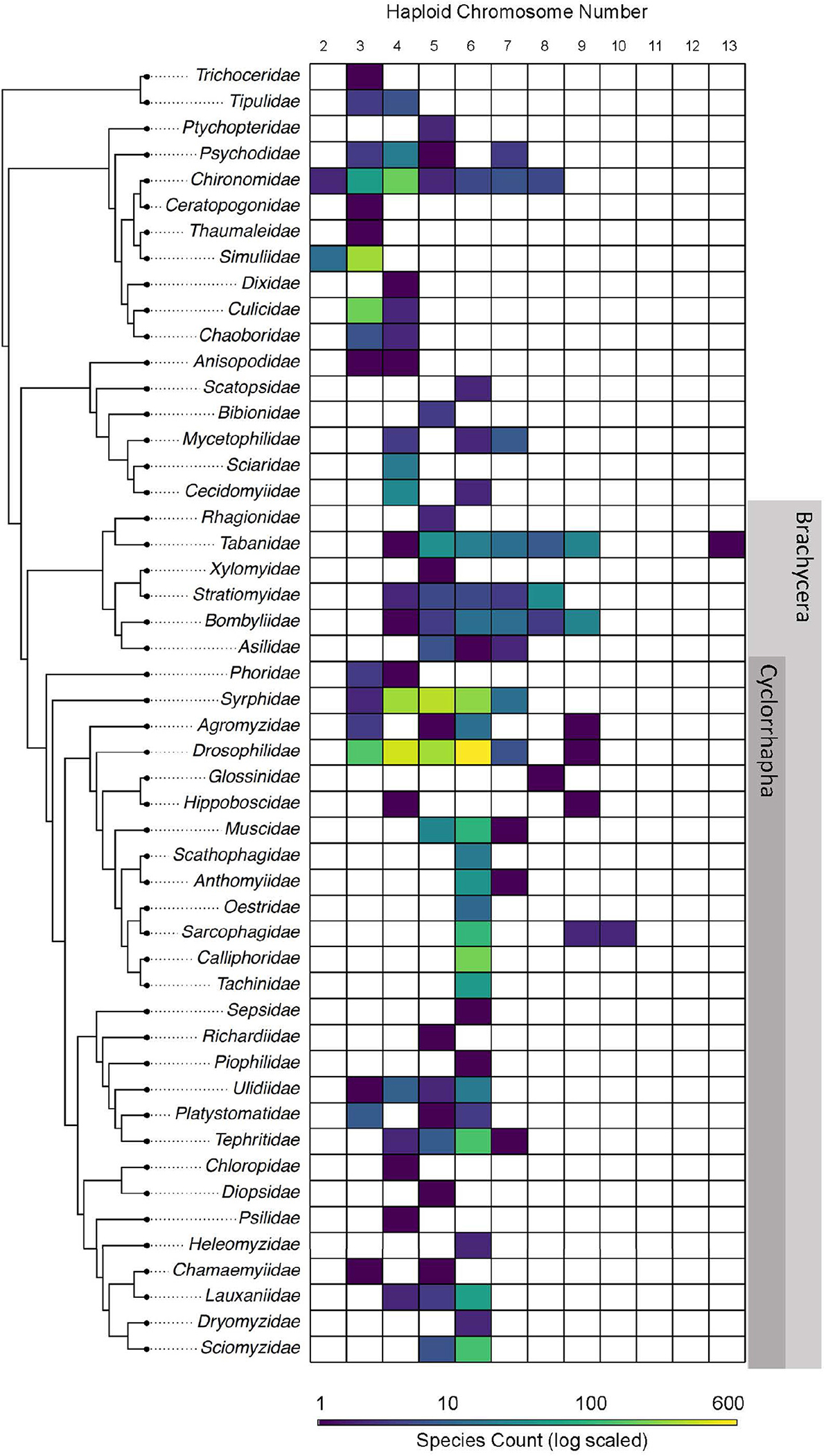
Phylogenetic distribution of haploid chromosome count in Diptera. In each row the number of species with a specific haploid chromosome count is indicated by color. Note the color ramp is on a log scale to allow simultaneous visualization of families like Drosophilidae and Piophilidae which have orders of magnitude differences in the number of records available.

A total of 36% (1,244) of the records we found are from *Drosophila*. These records are available as part of the Diptera database but also the more focused *Drosophila* Karyotype Database. When examining all data in a phylogenetic perspective it appears some clades may have less stable karyotypes than others [phylogeny from Wiegmann et al. (2011)]. For instance, in Figure 1, we can compare two families like Simuliidae (237 records) and Chironomidae (181 records). Generally assuming one record per species, that suggests we cover approximately 14% of the 1,700 estimated species in Simulidae and merely 1–2% of the estimated 10,000 species in Chironomidae. We might expect Simuliidae to exhibit more variation in chromosome number since we have considerably more records for this group, both in raw number and taxon representation. However, our synthesis of all available data indicates Chironomidae, with just 181 records, has haploid numbers ranging from two to eight, while Simuliidae has only haploid counts of two and three, despite more extensive sampling, suggesting that rates of karyotype evolution are accelerated in Chironomidae relative to Simuliidae. Combining this data in the future with species level phylogenies would allow the application of probabilistic models of chromosome evolution (Zenil-Ferguson et al., 2018; Blackmon et al., 2019). This approach may identify which clades have more or less stable genome structure, and the underlying causes of these differences in the tempo of evolution and potential correlations with speciation rates.

Our database confirms that the vast majority of species (96%; 3,341) have a haploid chromosome count of six or less, suggesting a strong conservation of the six Muller elements (Figure 1). When visualizing the distribution of chromosome number on the phylogeny and focusing on records with six or fewer haploid chromosomes, it is apparent that species within Cyclorrhapha consistently have higher average chromosome number than those of earlier branching clades (Figure 1). This pattern suggests that chromosome number may have “settled” at the level of the six Muller elements in the cyclorrhaphous Diptera from an ancestral condition with fewer chromosomes. In fact, when viewed phylogenetically, chromosome number seems to have increased in steps, with the earliest branching Brachycera having chromosome numbers intermediate between Cyclorrhapha and earlier branching families, like Simuliidae and Culicidae. Further comparative phylogenetic approaches are necessary to validate this pattern of chromosome numbers in the phylogeny of Diptera.

While there is exceptional conservation of the six Muller elements in most of the Diptera phylogeny, there are a number of species which exceed this number. The 133 species with more than six chromosomes are not phylogenetically clustered; rather, 16 families (~25% of all families with data) have at least one species with greater than 6 chromosomes. Three families of orthorraphous dipterans (Bombyliidae, Tabanidae, and Stratiomyidae) have more than 20 species with greater than six chromosomes. It has been hypothesized that the paucity of species with more than six chromosomes in Diptera is driven by reliance on retrotransposons (evidence from *Drosophila* species) or tandemly repeated sequences (evidence from lower Diptera) to protect chromosome ends (Diptera lack telomerase) (Mason et al., 2011; Sved et al., 2016). Using our database provides a straightforward workflow to identify several distantly related species all of which overcame this apparent threshold on chromosome number. In fact, one of these species *Hermetia illucens* (black soldier fly) has a haploid chromosome number of seven. This species was recently sequenced (Generalovic et al., 2021) combining this with additional species could help researchers understand how species can remodel their genome in the absence of telomerase.

**FIGURE 2 |:**
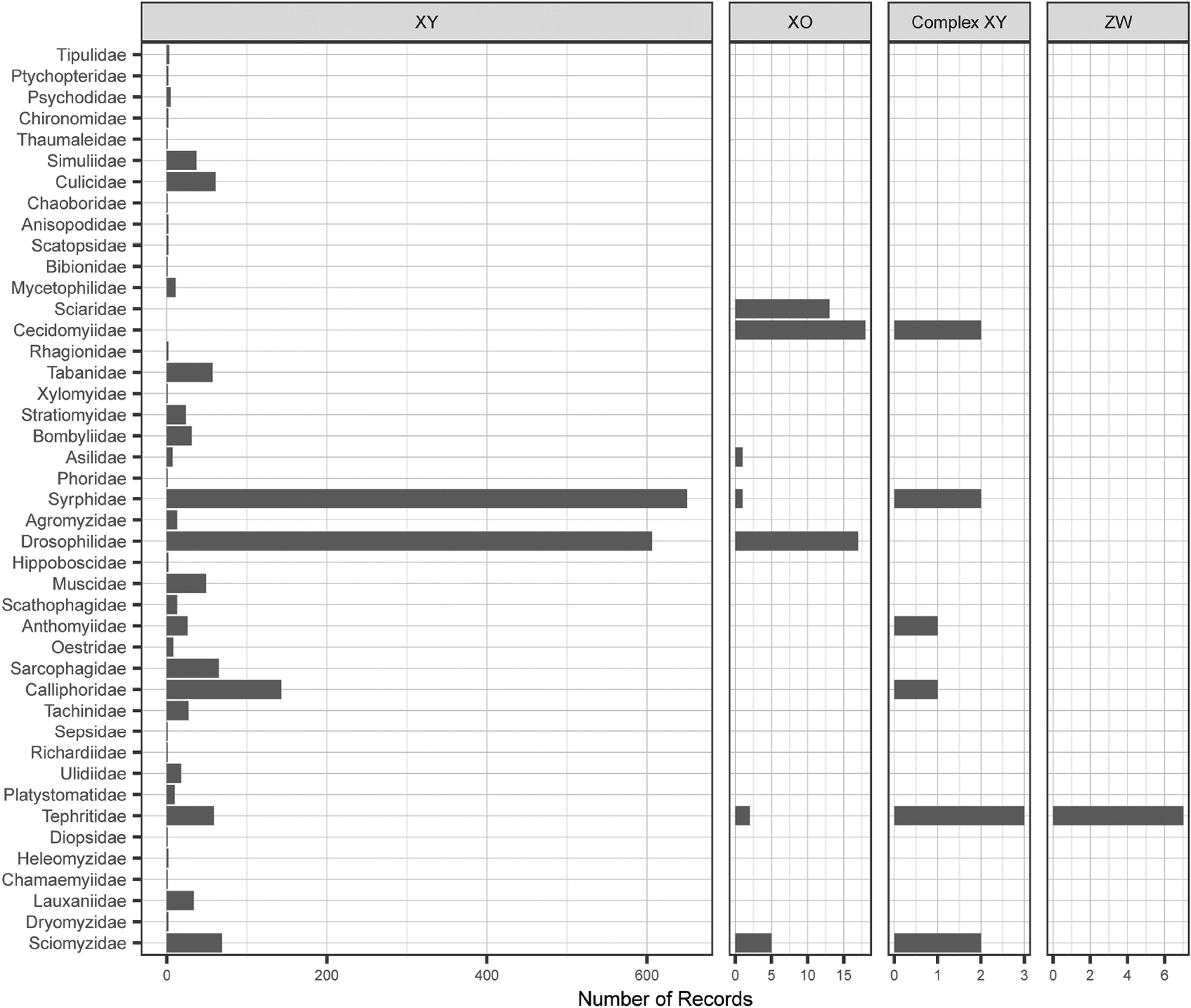
Distribution of sex chromosome systems across families of Diptera. The order of families is the same as in Figure 1. Note scales on horizontal axis vary in size to allow visualization of rare sex chromosome systems.

Our database contains sex chromosome system information for 2,128 species (Figure 2). The vast majority of species have an XX/XY system (2,053 species). The remaining records include 11 multi-XY(X_1_X_2_Y, X_1_X_2_Y_1_Y_2_, XY_1_Y_2_), 57 XO, and 7 ZW systems. Loss of the Y chromosome, though rare, is relatively widespread. The 57 species with XO sex chromosome systems are spread across seven families with the most species (18) found in Cecidomyiidae. Interestingly Cecidomyiidae exhibits striking variation in sex chromosome systems. One species, *Phytophaga destructor*, exhibits homomorphic sex chromosomes likely an indication of sex chromosome turnover. Two other species (*Mayetiola destructor* and *M. hordei*) possess a multi sex chromosome system (X_1_X_1_X_2_X_2_/X_1_X_2_Y). These systems are usually found in cases where an autosome has fused to the Y chromosome (although fissions of the X chromosomes could presumably result in a X_1_X_2_Y system) in a species that was ancestrally XX/XY (a state unlikely to be ancestral in this group since 18 of 21 species are XX/XO). Its presence in these two species is even harder to explain because there is no reduction in its haploid number in comparison to related species; both species in the genus *Mayetiola* have a haploid number of four as do 17 of the other 19 species in this family.

The distribution of sex chromosome systems also highlights the importance of the family Tephritidae. While this family is relatively unremarkable when we consider haploid number (range from 4 to 7), sex chromosome systems in this group appear to be highly unstable. In this family, we have sex chromosome information for 71 species, yet we find all of the diversity of systems exhibited by the entire order of Diptera. The XX/XY system is still the most common, with 59 species spread wide taxonomically across 14 genera. The next most common is ZZ/ZW sex chromosome system, with seven species in five genera, which are likely from single origin (Frias, 1992). However, in the Tephritis genus, there is a species with an XO system, showing that even sister species can have different heterogametic sexes. We also find complex XX/XY sex chromosome systems in three species (two in the genus *Anastrepha* and one in the genus *Rhagoletis*). Finally, the XX/XO sex chromosome system is found in just two species *Rhagoletis meigeni* and *Tephritis arnicae*.

In insects, it has been hypothesized that complex sex chromosome systems often arise through fusions of autosomes with sex chromosomes (Blackmon and Demuth, 2015b). In fact, a recent study of Polyneoptera showed that species with complex sex chromosome systems have systematically lower numbers of autosomes (Sylvester et al., 2020). However, synthesizing all of our collected records we find Diptera with complex sex chromosome systems actually have a higher haploid number (mean of 5.5) than do species with a simpler and likely ancestral XX/XY sex chromosome system (mean of 5.2). A recent study of patterns of sex chromosome autosome fusions in *Drosophila* has suggested that the generation of these complex sex chromosome systems may be limited in species with achiasmatic meiosis (Anderson et al., 2020). However, at most this is only a partial explanation as not all Diptera possess achiasmatic meiosis.

Despite reductions in the cost of sequencing, we are still far from being able to sequence and *de novo* assemble all genomes. Moving forward, we believe one of the most powerful uses of this data will be to identify species whose genomes promise to be most useful in investigating the dynamics of genome structure evolution.

## Figures and Tables

**TABLE 1 |:** Traits recorded in karyotype databases.

Trait	States
Taxonomy	Family/Subfamily; Tribe; Genus; Species
Haploid Chromosome Number	2–13
Chromosome Shapes	Submetacentric; Metacentric; Subtelocentric; Telocentric; Subacrocentric; Acrocentric; Dot; Sex Chromosome Shape
Chromosome Shapes (*Drosophila* specific)	V for Metacentric; J for Submetacentric; Rod for Telocentric/Acrocentric
Sex Chromosome System	Complex XY; XO; XY; ZW; Monogenic
References	Citation for the sources of data included in the database

## Data Availability

Publicly available datasets were analyzed in this study. This data can be found here: www.karyotype.org.

## Footnotes

1
https://library.tamu.edu/

2
https://www.lib.utexas.edu/

3
http://treeofsex.org/
